# What type of mattress should be chosen to avoid back pain and improve sleep quality? Review of the literature

**DOI:** 10.1186/s10195-021-00616-5

**Published:** 2021-12-08

**Authors:** Gianfilippo Caggiari, Giuseppe Rocco Talesa, Giuseppe Toro, Eugenio Jannelli, Gaetano Monteleone, Leonardo Puddu

**Affiliations:** 1 Orthopaedic and Traumatology Department, Università degli Studi di Sassari, 07100 Sassari, Italy; 2 Orthopaedic and Traumatologic Clinic, University of Perugia, Santa Maria della Misericordia Hospital, 06129 Perugia, Italy; 3 Department of Medical and Surgical Specialties and Dentistry, University of Campania “Luigi Vanvitelli”, 80138 Naples, Italy; 4 Department of Orthopaedics and Traumatology, Fondazione Policlinico IRCCS San Matteo, University of Pavia, 27100 Pavia, Italy; 5 Orthopaedic, Trauma and Spine Unit, Department of Basic Medical Sciences, Neuroscience and Sense Organs, School of Medicine, AOU Policlinico Consorziale, University of Bari “Aldo Moro”, 70100 Bari, Italy; 6 Orthopaedic and Traumatology Department, Rovereto – Arco Hospital, 38068 Rovereto, Italy

**Keywords:** Mattress, Back pain, Sleep quality, Sleep, Quality of life

## Abstract

Energy spent during daily activities is recuperated by humans through sleep, ensuring optimal performance on the following day. Sleep disturbances are common: a meta-analysis on sleep quality showed that 15–30% of adults report sleep disorders, such as sleep onset latency (SOL), insufficient duration of sleep and frequently waking up at night. Low back pain (LBP) has been identified as one of the main causes of poor sleep quality. Literature findings are discordant on the type of mattress that might prevent onset of back pain, resulting in an improved quality of sleep. We conducted a systematic literature review of articles published until 2019, investigating the association of different mattresses with sleep quality and low back pain. Based on examined studies, mattresses were classified according to the European Committee for Standardization (2000) as: soft, medium-firm, extra-firm or mattresses customized for patients affected by supine decubitus. A total of 39 qualified articles have been included in the current systematic review. Results of this systematic review show that a medium-firm mattress promotes comfort, sleep quality and rachis alignment.

## Introduction

Human beings usually spend around a third of their lifetime sleeping [1], even though this enables individuals to satisfactorily exploit the remaining two-thirds of the day only if sleep was free from disturbances or interruptions. In the past years, an increasing number of people have been complaining of sleep disorders [2].

Although optimal sleep duration is between 7 and 8 h per day, it has been highlighted that a reduction in sleeping hours has occurred, due to work habits or overall changes in lifestyle. A reduction in sleep hours or sleep quality inevitably has negative impacts on individuals’ health, as well as on life and mood quality [3].

Low back pain should be taken into consideration among factors that reduce sleep quality.

Although several studies acknowledge the significance of mattresses for sleep quality, there is no common agreement on the optimal design of a mattress to alleviate or prevent cervical or low back pain. Detrimental effects on health led us to analyse which mattress might act as a solution to these problems [4].

Mattress firmness seems to play a leading role as different studies show that medium-firm surfaces might effectively reduce pain in individuals complaining of back pain [5].

An increasing number of companies are promoting their mattresses claiming that they might be able to improve sleep quality and quality of life as a consequence, pretending that their mattresses are “orthopaedic mattresses” with therapeutic properties. However, such claims are not supported by enough evidence.

Several studies in the literature aimed to assess which could be the best mattress to prevent back pain and improve sleep and life quality; however, the lack of univocal findings suggests that additional research on this issue is warranted. Therefore, the aim of this review is to evaluate available studies so as to understand which mattresses can effectively reduce back pain or prevent its onset. In this way, healthcare professionals will be able to recommend to patients the correct type of mattress to limit or avoid back pain symptoms, thus providing them with benefits in terms of life quality.

## Materials and methods

Studies carried out between 2000 and 2019 have been included in the current review. Mattresses analysed in the studies examined by our research group have been classified according to the European Committee for Standardization (2000) as soft, medium-firm, extra-firm or customized to avoid supine decubitus.

The databases searched include the Cochrane Library, PubMed (Digital Biomedical Archives and Health Sciences of the US National Institutes of Health), Google Scholar, Web of Science and Scopus.

The selection of search terms was tailored to match the search tools of each database, using MeSH terms to search in the databases Science Direct, PsycINFO, EMBASE, PubMed, Google Scholar, Web of Science, Scopus and Cochrane Collaboration. The following terms were used as descriptors: mattress and ergonomics, mattress and pain, mattress and vertebral column, mattress and sleep, mattress and quality of life.

Articles were sourced by searching the databases using the search strategy, or by searching for “similar articles”. Inclusion criteria for the articles were publication in English, being published between the years 2000 and 2019 and studies conducted on adults over 18 years of age.

The first screening of articles was carried out by reading the titles and abstracts; those that had no relation to the subject, considering the inclusion and exclusion criteria, were excluded. A total of 323 potentially relevant papers were identified in the five investigated databases; these articles were selected by eliminating those with titles that did not correspond with the search, for example concerning certain categories (athletes, children, hospitalized adults), or were aimed at the study of certain pathologies (sudden death, ulcers, asthma, and other pathologies not related to our study), excluding 194 duplicates. After abstract analysis and reading, 70 articles were selected for full reading, after which 31 articles were excluded. Articles were excluded if they did not account for any biomechanical measurements or investigations; for example, some excluded articles only dealt with posture behaviour, cardiovascular and pulmonary measurements, questionnaires, temperature and humidity. Articles on medical-use mattresses, particularly those describing anti-pressure sore mattresses, were also excluded.

**Fig. 1:**
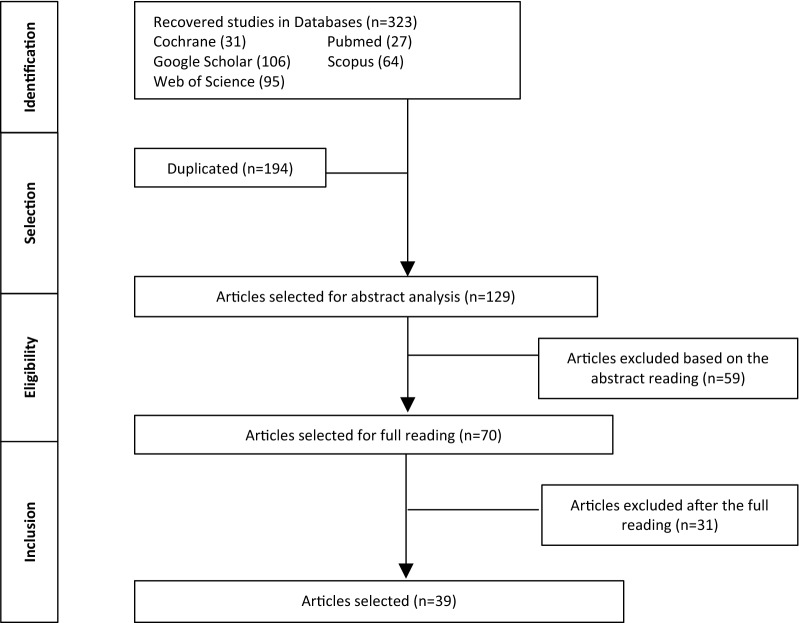
Flow selection of articles, according to the PRISMA

There was a total of 39 articles for analysis. The process of selecting studies is shown in Fig. 1, using a Preferred Reporting Items for Systematic Reviews and Meta-Analyses (PRISMA) flowchart.

A data extraction table was designed to collate information from the studies. For the analysis of the studies, an instrument composed of the following items was used for obtain data: author name, study type, research designs, levels of evidence (Melnyk & Fineout-Overholt, 2005), populations, samples, results and conclusions. These research characteristics are presented in Table 1.

**Table 1:** Summary of articles on the effects of different types and designs of mattresses, on sleep quality and pain reduction

Author (year)	Study design	Aim	Materials and methods	Conclusion
Kovacs et al. [9]	Randomized, blinded, controlled trial	Evaluation of effects of mattress firmness on patients affected by chronic low back pain	Level of evidence: I 313 participants (155 medium-firm mattresses allocated to patients and 158 firm mattresses allocated to patients) Patients with ≥ 3 months chronic back pain while lying in bed or on rising VAS score used to measure level of pain symptoms Roland Morris questionnaire administered to assess degree of disability	Although no statistically significant pain reduction was reported among groups observed, the study shows that patients with chronic low back pain can benefit from mid-firm mattresses
McCall et al. [6]	Randomized controlled trial	Comparison between a traditional and an anti-decubitus mattress, with seven different pressure areas for 2 weeks	Level of evidence: II 12 participants Asymptomatic Use of VAS score to measure level of pain symptoms Actigraphy and pressure mapping	No statistically significant difference between the two mattresses in any of the measurements obtained; however, the anti-decubitus mattress reduced the number of high-pressure points
Bergholdt et al. [10]	Randomized single-blinded clinical trial	Comparison of three different types of mattresses: waterbed (Akva), memory foam mattress (Tempur), firm mattress (Futon Innovation) for 1 month	Level of evidence: II 160 participants Symptomatic Danish COBRA questionnaire administered to measure pain symptoms levels and activities of daily life (ADLs)	Water mattresses and foam mattresses showed best results for low back pain
Jacobson et al. [14]	Controlled trial	Comparison of sleep quality based on mattress firmness	Level of evidence: III 22 participants Symptomatic VAS score used to evaluate low back pain, stiffness, shoulder pain and sleep quality	Patients reported improvement of low back and shoulder pain
Jacobson et al. [5]	Controlled trial	Assessing new mid-firm mattresses systems	Level of evidence: III 59 patients Used two questionnaires (VAS score to evaluate pain symptoms, discomfort, rachis stiffness, sleep quality; and another questionnaire related to potential stress, anxiety and irritability conditions) Asymptomatic	Mid-firm mattresses ensure more satisfactory levels in terms of sleep, comfort and pain symptoms
Jacobson et al. [7]	Non-randomized controlled trial	Influence on sleep quality through comparison of new mattresses and personal mattress systems	Level of evidence: III 59 patients (29 male and 30 female) Asymptomatic Two-phases study Evaluation of sleep quality through personal mattresses for 28 days Evaluation of sleep quality on a new mattress system for 28 days Use of VAS scale to assess quality of sleep, comfort and efficiency of the mattress system adopted and of pain symptoms involving low back, shoulders and rachis stiffness	New mattress systems can significantly improve sleep variables considered and quality of sleep arising from prompt replacement of mattress system components
Jacobson et al. [8]	Controlled trial	Comparison of sleep quality when using a mid-firm mattress and patient’s own mattress	Level of evidence: III 59 patients (29 male and 30 female) Asymptomatic Use of VAS scale to evaluate pain symptoms and sleep quality Questionnaire formed by 32 questions related to potential stress conditions	Mid-firm mattresses are given a positive evaluation as far as reduction of low back pain and sleep quality improvement are concerned
Jacobson et al. [17]	Controlled trial	Comparison of sleep quality by using subjects’ own mattresses for 3 weeks and the prescribed mattress for 12 weeks	Level of evidence: III 27 participants Symptomatic Use of VAS scale to assess pain symptoms and sleep quality	Significant improvement in sleep quality and pain symptoms through new technologies-based mattress prescribed
Monsein et al. [11]	A-B-A design	Pain reduction and sleep quality effects induced by individuals’ own mattress and an air-adjustable mattress placed on the topper	Level of evidence: III 90 participants Patients complaining of low back pain Analysis of data linked to participants’ own mattress Analysis of the second type of mattress for 28 days Results evaluation conducted through Short Form (SF) 36 health survey Epworth Daytime Sleepiness Scale, and VAS	Improvement of sleep quality and low back pain following use of air mattress
Price et al. [12]	Pilot study	A-B design prospective study to evaluate sleep quality by using an air mattress system (Repose; Frontier Therapeutics Ltd, Blackwood, South Wales)	Level of evidence: III 19 patients Patients with chronic low back pain and sleep disorders Use of a VAS evaluation chart on sleep quality	Statistically significant results found as a consequence of air mattress system use
Bader and Engdal [16]	Randomized controlled trial	Comparison between soft and firm mattress systems and influence on sleep quality	Level of evidence: II Ten participants Asymptomatic Evaluation chart used to assess pain, stress and fatigue Use of polysomnography to evaluate sleep quality	No statistically significant result arising from comparison between two mattress systems
Lahm and Iaizzo [13]	Non-randomized controlled trial	Evaluation of cervical dorsal column orientation and its effect on sleep quality using three mattress systems with adjustable air chambers	Level of evidence: III 29 participants (15 male, 7 female) Asymptomatic Assessment of EMG activity, heart rate, blood pressure, subjective comfort levels and data on column alignment on different pressure degrees of the mattress system Questionnaire related to subjective opinions on sleep quality	Although mattress inflation pressure induced significant changes on spinal alignment, these had limited physiological consequences. However, such data provide essential information to evaluate similar associations in a symptomatic population (acute/chronic low back pain and cervical pain)
López-Torres et al. [21]	Non-randomized controlled trial	Evaluation of sleep quality taking into consideration four types of mattresses: spring mattress, latex, polyurethane, two-layer firmness system	Level of evidence: III 75 participants Asymptomatic Questionnaire regarding structural and morphological qualities of the mattress (firmness and softness)	Results of comparative analysis were correlated to differences of objective properties such as pressure distribution and objective firmness. Consequently, results on morphological and structural characteristics of mattress systems analysed (firmness and softness) were directly proportional to comfort reported by patients analysed
Raymann et al. [19]	Experimental	Evaluation of skin temperature during sleep	Level of evidence: VI 24 participants (8 young adults, 8 old asymptomatic subjects and 8 symptomatic subjects) Asymptomatic patients and patients with insomnia Proximal and distal skin temperature manipulation via thermal suit between 12:00 and 6:00 AM. Cycling between 31.7 °C ± 0.1 °C in “cool” and 34.5 °C ± 0.1 °C in “warm”. Total length of test was 4 days. Day 1 was spent sleeping at home. Day 2 was spent sleeping in the laboratory. Day 3 was spent sleeping at home. Day 4 was spent sleeping in the laboratory. Subjects wore the thermal suit on days 2 and 4	Temperature check in bed through soft manipulation might have a strong clinical impact on sleep alterations, especially in the elderly who are not able to effectively respond to temperature variations
Tonetti et al. [1]	Actigraphic study	Evaluation of sleep quality comparing the use of a spring mattress and the Myform system for 2 weeks	Level of evidence: VI 28 participants (14 male, 14 female) Asymptomatic Mini Sleep Questionnaire and Hassles Scale adoption Evaluation of sleep parameters: sleep efficiency, insomnia latency and motor activity	Sleep quality improves thanks to Myform, but this result does not seem statistically significant
McCall et al. [6]	Controlled trial	Evaluation of sleep quality using an intelligent mattress system. Comparison between standard and dynamic configuration	Level of evidence: III 11 participants Asymptomatic Use of polysomnography to assess sleep Karolinska Sleepiness Scale, Profile of Mood State Subjective evaluation regarding sleep quality	Active control system with dynamic configuration resulted in increased sleep quality. Participants perceived fewer awakenings and awakenings were shorter with the active control system with dynamic configuration
Verhaert et al. [27]	Non-randomized controlled trial	Evaluation of an ergonomic mattress system and its effect on sleep	Level of evidence: III 17 participants Asymptomatic Use of different questionnaires [VAS (0–20),Karolinska Sleepiness Scale, Cox’s Stress/Arousal, Adjective Check List, the fatigue scale of Profile of Mood] Polysomnography to objectively evaluate sleep quality Video-recording aids	The effect of bed design on sleep cannot be fully evaluated just comparing two sleep systems. A relaxed sleep system has a negative effect on sleep quality for people who spend most of the time in a lateral sleep position. Individuals who sleep in lateral position also spent a significantly shorter time in REM compared with the standard condition
Park et al. [18]	Multicenter controlled trial	Evaluation of the relations between the characteristic of mattress, anthropometric features, body pressure distribution and spinal curvature and to examine overall relations between the comfort and features of mattress. Six materials, i.e. three kinds of cotton, felt, sponge and elastic cotton, were used as pad materials. Springs were 3-pitch, 4-pitch or 5-pitch	Level of evidence: III 18 participants (9 male, 9 female) Asymptomatic 3D measurement of vertebrae column at C7, T1, T3, TT, Tg, T11, L2, L5/S1 and of sacrum and coccyx prominences Pressure measurement at hips and shoulders through sensors The subject fills out six evaluation charts that uses seven-point scales (divided into two sections) about the degree of satisfaction and physical features	The best mattress was the mattress in which the spinal curvature in lying position was most similar to that in standing. The firmness had to be extended to increase patient comfort
Price et al. [12]	Pilot study	Prospective study AB design about the use of an air flotation mattress for 4 weeks overlay in patients with chronic pain	Level of evidence: III 19 participants Patients with chronic pain symptoms and sleep alterations Evaluation about self-reported changes in sleep quantity and frequency of sleep disturbance, and about self-reported changes in pain and use of analgesia	This study reported statistically significant results about improvement in sleep and pain after 4 weeks with the use of a new (low-pressure inflatable) overlay mattress
Shen et al. [36]	Randomized control trial	Comparing 18 different types of spring mattresses (5 different spring cores, 14 different top comfort combination layers)	Level of evidence: II Eight participants Asymptomatic Polysomnography [electrocardiogram, electrooculogram, electroencephalogram, electromyography (EMG)], actigraphy (sleep/wake behaviour), body movements Questionnaire about level of fatigue of body parts before sleep, discomfort, sleepiness and pain after sleep	Morphological and structural features of the mattress influence sleep quality Sleep quality depends on postural characteristics Subjective opinion on mattress system characteristics
Lee et al. [31]	Randomized control trial	Study of the effects of the type of mattress on sleep quality by measuring the temperature of the skin, using a subjective mattress evaluation system and through the use of a polysomnogram	Level of evidence: II 16 participants (age range 20–30 years) Asymptomatic Personal recordings about sleep quality Polysomnography data, skin temperature	To ensure efficient sleep quality, a mattress must guarantee the best support for the spine, maintain constant body temperature and reduce body movements during sleep
DeVocht et al. [4]	Controlled trial	Objective, biomechanical comparison of four “top of the line” mattresses from four different manufacturers (mattress A, Perfect Contour Extraordinaire Dorchester by King Koil; mattress B, Beautyrest Calibri Firm by Simmons; mattress C, Posturepedic Afton Plush by Sealy; mattress D, Perfect Sleeper Southdale by Serta)	Level of evidence: III 18 patients (all male) Asymptomatic Two different measurements (pressure distribution during the supine position and evaluation of the degree of spinal distortion induced when in the side posture position)	The pelvic region had higher pressure values when compared with the thoracic region. The least amount of pressure was seen in mattress A (Perfect Contour Extraordinare Dorchester by King Koil), and the highest pressure was seen in mattress D (Perfect Sleeper Southdale by Serta). Mattress D also demonstrated the lowest level of spinal distortion
Leilnahari et al. [26]	Controlled trial	Evaluation of pressure exerted on the column based on the degree of mattress firmness: soft (polyurethane foam), firm, and custom-made mattresses	Level of evidence: III 25 participants (all male) Asymptomatic Comparison between pressure exerted on the column (through sensors placed on spinal processes) both in lateral and routine position	The results showed a significant difference in the *π*-P8 angles between soft and custom-made and soft and firm mattresses at the *p* = 0.001 level and between firm and soft mattresses at the *p* = 0.05 level A custom-made mattress can be effective for heavier patients. The stiffness of the mattress influences the forces exerted on the spine
Normand et al. [30]	Quasi-experimental	Evaluation of six different conditions using three types of surfaces (no mattress, 8 cm of foam, 14 cm latex mattress of medium density)	Level of evidence: III Ten participants Asymptomatic Assessment of pressure distribution on the thorax, pelvic and low back areas (Tekscan pressure sensor) in supine position for 30 s	Use of a low back support led to a homogeneous pressure on the thorax, low back and pelvis in supine position
Chen et al. [22]	Randomized cross-over, single-blinded controlled trial	To investigate the influence of mattress firmness on body contact pressure and sleep quality	Level of evidence: II 16 healthy males (aged 20–45 years) Sleeping posture: supine lateral Mattress characteristics: (1) Plank springs (2) With supporting layer and pillow top made of palm fibre (3) 3D structure made of foam rubber and plant fibre, with supporting layer, intermediate layer finely fitting the shape of the human body, and pillow top. (4) Independent springs Methods: (1) ABW body pressure measurement system; (2) ALICELE PSG polysomnography; (3) questionnaire, yes/no questions on hardness, comfortability, and difficulty to fall asleep Measurement: (1) Body-mattress contact pressure (2) Sleep quality/polysomnography (3) Subjective feedback	The results reveal that a mattress with an intermediate level of contact pressure led to better sleep quality
Denninger et al. [15]	Design process, validation of simulation (deviation)	Measurement of body dimensions, body mass distribution and force compression curve	Level of evidence: VI Sleeping posture: lateral Mattress characteristics: custom-made mattress consisted of rows and columns of PU foam (extra-firm Q41) cubes with hollow ellipsoidal cavities. Cube dimensions were customized according to spinal curvature and body weight portion Methods: (1) POWERSHOT A610 camera; (2) custom-made apparatus with load cells; (3) ANSYS, finite element method; (4) Optotrak 3020 optical measurement system Measurement: (1) Body dimensions (2) Body mass distribution (3) Force–compression curve of foam cubes loaded with body volume slice	A design process comprising a look-up table of human–mattress interaction predicted by simulation was established. The design of a customized mattress with different cube cavity dimensions could be defined together with the input of body properties. Validation showed a load distribution within a 10% average deviation from the expected distribution; spine alignment was within a distance of ± 3% shoulder width from the expected spine curvature
Deun et al. [37]	Repeated measures, non-randomized controlled trial	Investigation of sleep quality induced by an active-control bedding system that autonomously alters stiffness distribution according to the estimated spinal alignment, as compared with the inactive mode of this system	Level of evidence: III Three subjects (one female, two male) with non-specified age Sleeping posture: Lateral Mattress characteristics: Custom-made mattress consisted of rows and columns of PU foam (extra-firm Q41) cubes with hollow ellipsoidal cavities. Cube dimensions were customized according to spinal curvature and body weight portion Eleven healthy subjects (five female, six male) aged 20–28 years, mean age 21.2 ± 3.2 years Sleeping posture: No control, postures were detected and estimated Mattress characteristics: Dynasleep, mattress equipped with indentation sensors and adaptive actuator spring pockets. (1) Actuator inactive and (2) actuator active induced different stiffness in eight zones to optimize spinal curvature based on the results of indentation measurements Measurements: (1) body surface contour; (2) sleep quality/polysomnography; (3) spinal curvature; (4) subjective feedback Methods: (1) IKÉLO optical measurement system; (2) dream system, polysomnograph; (3) indentation sensors embedded in Dynasleep mattress (spinal curvature was simulated and estimated by indentation using a human model personalized based on the results of body contour measurements); (4) questionnaires: Karolinska sleepiness scale, profile of mood state, stress/arousal adjective checklist, activation/deactivation adjective checklist	When active control mode was used, sleep quality was significantly improved, as revealed by polysomnographic analysis and subjective feedback
Esquirol Caussa et al. [28]	Recommendation model, validation of somatotype model (correlation)	Design and validation of an automatic multimodal somatotype determination model to automatically recommend mattress–pillow topper design combinations	Level of evidence: VI First pilot test: six subjects, age/gender not specified; Second pilot test: 50 subjects (28 female, 22 male) aged 18–93 years, mean 34.2 years; final study: 151 subjects (60 female, 91 male) aged 4–94 years, mean 34.43 years; re-analysis study: 117 subjects (75 female, 42 male), aged 4–93 years, mean 33.82 years Sleeping posture: Supine Mattress characteristics: (1) Soft, density 2.75 kPa*; (2) neutral/soft, density 3.0 kPa; (3) neutral, density 3.3 kPa; (4) neutral/hard, density 3.8 kPa; (5) hard, density 4.4 kPa. Three types of toppers (DORMITY): (1) soft, density 1.1 kPa; (2) medium, density 1.6 kPa; (3) hard, density 2.1 kPa. Three types of pillows of different densities (45 combinations) Measurements: (1) Body dimensions; (2) body–mattress contact pressure Methods: (1) Kinect camera and tape; (2) surface with integrated pressure capacitive sensors	Validation of somatotype models demonstrated a high correlation index compared with real data: more than 85% in height and body circumferences; 89.9% in weight; 80.4% in body mass index; and more than 70% in morphotype categorization
Lee et al. [24]	Mixed factorial design (gender, body regions, duration), non-randomized controlled trial	Analysis of body pressure and perceived level of pain for different genders, body regions and durations of supine lying	Level of evidence: III Ten healthy subjects (five female, 5 male), age mean 29.1 ± 3.2 years Sleeping posture: Supine Mattress characteristics: Subjects’ existing mattress Measurement: (1) Body–mattress contact pressure; (2) subjective feedback Methods (1) Body pressure measurement system; (2) questionnaires: pain score using visual analogue scale, faces pain rating scale, Iowa pain thermometer	Head regions experienced significantly higher pain scores and pressure intensities; lower back was not too high in pressure intensity but featured the second-highest pain score; the back and pelvic girdle showed a significant difference between males and females on the pain score; pain appeared in all body regions after 10 min and progressed as time increased
Lee et al. [23]	Repeated measurements, non-randomized controlled trial	Comparison of body pressure and perceived level of pain between the floor and mattress for different durations of supine lying	Level of evidence: III Ten healthy subjects (five female, five male), age mean 29.1 ± 3.2 years Sleeping posture: Supine Mattress characteristics: (1) Floor; (2) mattress Measurement: (1) Body–mattress contact pressure; (2) subjective feedback Methods: (1) Body pressure measurement system; (2) questionnaires: pain score using visual analogue scale, face pain rating scale, Iowa pain thermometer	Head regions featured a significantly higher-pressure intensity; the pain scores of all body regions except for legs were significantly higher for the floor condition; the pain score of the floor condition significantly increased at 1 min compared with those of the mattress group
Leilnahari et al. [26]	Design process, repeated measurements, non-randomized controlled trial	Design of a customized mattress with different zonal elasticity that can achieve optimal spinal alignment; comparison of spinal alignment achieved by firm, soft and custom mattresses	Level of evidence: III 25 male students Sleeping posture: lateral Mattress characteristics: Spinal deformities: lateral. (1) Soft mattress (polyurethane foam and a layer of memory foam; (2) firm mattress; (3) custom-made mattress with different regional stiffness based on neutral spine alignment predicted by the musculoskeletal model. The mattress was made of a combination of PU and spiral pressure springs with different wire diameters Measurements: spinal curvature Methods: (1a) DCR-TRV356E cameras; (1b) BRG.LIFEMOD2007, musculoskeletal modelling (spinal curvature was simulated and estimated by modelling and validated by captured images)	The customized mattress with different zonal elasticity afforded better spinal alignment (least π-P8), followed by firm and soft mattresses
López-Torres et al. [21]	Non-randomized controlled trial, correlation	Comparison of perceived firmness, usability and comfort between young and elderly people; investigation of the correlation between subjective ratings and results of objective measurements (pressure distribution and objective firmness)	Level of evidence: III 19 young subjects (9 female, 10 male), age mean 28 ± 3 years (female), 26 ± 3 years (male); 56 elderly subjects (34 female, 22 male), age mean 67 ± 5 years (female); 70 ± 6 years (male) Sleeping posture: Three-step testing procedure: (1) seated position; (2) supine; (3) roll onto one side. Four mattresses were selected from 17 samples to cover the full range of firmness Mattress type: Four mattresses were selected from 17 samples to cover the full range of firmness Measurement: (1) Mannequin-mattress contact pressure; (2) subjective feedback Methods: (1) PLIANCE 19 P body pressure measurement system; (2) questionnaire: perceived firmness with hands, buttocks, in supine/lateral posture; difficulties in rolling over and getting up; four-point grading in comparing overall comfort	No perception differences between the young and the elderly were found. Significant correlations were found between increments in objective firmness and perceived firmness (positive); increments in average pressure and perceived firmness (positive); increments in objective firmness and average pressure were associated with increments in overall comfort and reductions in rolling difficulty
Low et al. [25]	Randomized cross-over, single-blinded controlled trial	Comparison of the body contact pressure profile of different mattresses in three different postures	Level of evidence: II 20 young healthy subjects (10 female, 10 male), age: not specified Sleeping posture: Supine lateral prone Mattress characteristics: (1) Delight, latex foam mattress; (2) Masterfoam 1000, high-density PU foam mattress Measurements: body–mattress contact pressure Methods: (1) TEKSCAN 5400 N pressure mapping sensor	Compared with the case of a PU mattress, reduced peak pressure and a more even pressure distribution were observed for a latex mattress
Palmero et al. [29]	Recommendation model, validation for morphotype categorization (confusion matrix, correlation)	Development and validation of a somatotype determination model based on 3D RGBdepth imaging (Kinect) and automatic landmark points extraction; establishment of a recommendation model for mattress–pillow topper design combinations based on somatotype model and pressure analysis	Level of evidence: III 200 subjects (128 female, 72 male) aged 4–93 years, mean 33.82 ± 23.02 years Sleeping posture: supine Mattress characteristics: intermediate-density mattress Measurements: (1) body surface contour; (2) body–mattress contact pressure Methods: (1) Kinect camera; (2) in-house built capacitive pressure-sensitive mattress sensor	The system was capable of accurate categorization and achieved high correlation results with respect to manual measurement
Park et al. [31]	Design process, repeated measurements, non-randomized controlled trial	Development of an adjustable bed that regulates the height of eight mattress sectors and allows self-adjustment: comparison of adjustable bed and flatbed comfort ratings	Level of evidence: III 64 healthy subjects (35 female, 29 male) aged 25–50 years Sleeping posture: supine lateral prone Mattress characteristics: adjustable bed system with eight sectors that allowed the sector height to be controlled by subjects to achieve the most comfortable feeling; (1) without adjustment, (2) with adjustment Measurement: (1) body–mattress contact pressure; (2) subjective feedback Methods: (1) Self-assembled force-sensing resistor matrix; (2) questionnaire, five-point scale of comfortability in nine body regions (neck, shoulder, back, elbows, lumbar, hand/wrist, hip/thigh, knee, ankle)	Subjects preferred height adjustment in W-shape in supine and lateral postures, and in U-shape in lateral prone postures The adjusted height was significantly correlated with (a) the subjective rating and (b) the ratio of bed sector regional pressure and the total bed pressure
Verhaert et al. [27]	Repeated measurements, non-randomized controlled trial	Investigation of the effect of an active-control bedding system autonomously altering stiffness distribution according to the estimated spinal alignment and comparison to a sagging bedding system	Level of evidence: III 17 healthy subjects (8 female, 9 male), age mean 24.3 ± 7.1 years Sleeping posture: no control, biomechanical measurement on lateral posture only Mattress characteristics: Dynasleep, mattress equipped with indentation sensors and adaptive actuator spring pockets. (1) Actuator active, induced different stiffness in eight zones to optimize spinal curvature based on the results of indentation measurements; (2) manually adjusted actuator to simulate a sagging support (high stiffness at shoulder zone, low stiffness at the waist and hip zones) Measurement: (1) body dimensions; (2) body surface contour; (3) spinal curvature Methods: (1) calliper and tape; (2) IKÉLO optical measurement system; (3) indentation sensors embedded in Dynasleep mattress (spinal curvature was simulated and estimated by indentation using a human model personalized based on the results of body contour measurements)	The sagging sleep system negatively affected sleep quality in prone and lateral postures; the relationship between mattress design and sleep quality was affected by anthropometry and posture
Verhaert et al. [16]	Instrument design, validation (correlation)	Development of an estimation method for spinal alignment by integration of a personalized human model and mattress indentation measurements	Level of evidence: III 65 subjects (33 female, 32 male), age mean 27.3 ± 11.5 years. Validation: subgroup of 20 subjects (8 female, 12 male), age mean 22.9 ± 3.8 years Sleeping posture: Supine lateral prone Mattress characteristics: Dynasleep, mattress equipped with indentation sensors and adaptive actuator spring pockets. (1) Actuator active, induced different stiffness in eight zones according to anthropometric measurements and BMI; (2) manually adjusted actuator to simulate a sagging support Measurement: (1) Body dimensions; (2) body surface contour; (3) spinal curvature Methods: (1) Calliper and tape; (2) IKÉLO optical measurement system; (3) indentation sensors embedded in Dynasleep mattress (spinal curvature was simulated and estimated by indentation using a human model personalized based on the results of body contour measurements)	Good intraclass correlation (0.73–0.88) between estimated and measured angular spinal deformation was observed
Verhaert et al. [38]	Instrument design, validation (deviation), recommendation model	Estimation of spinal shape using a personalized anthropometric model and load–deflection characteristics of the mattress and bed base; presentation of a method to identify mattress bed base combinations with superior support properties	Level of evidence: III 18 subjects (9 female,9 male), age mean 28.5 ± 4.7 years Sleeping posture: Lateral three types of bed base: (1) homogeneous box-spring; (2) multi-zone slatted base; (3) multi-zone mesh base Mattress characteristics: (1) Multi-zone pocket spring mattress; (2) multi-zone latex mattress; (3) homogeneous PU foam mattress (nine combinations) Measurement: (1) Body surface contour; (2) body surface contour (for validation); (3) spinal curvature Instruments: (1) IKÉLO optical measurement system; (2) zSnapper 3D scanner; (3) spinal curvature was simulated and estimated based on the mass distribution of body portions and the human model personalized by body surface measurements and validated by 3D scanning	Estimation showed good correspondence (85%) in comparison with the validated spine shape in terms of score ranking
Verhaert et al. [39]	Mattress design process, randomized crossover single-blinded controlled trial	Presentation of an active control mattress system that can: (1) detect body movement and recognize sleep posture; (2) estimate the shape of the spine by combining indentation with human models; (3) based on indentation measurement and feedback, control the mattress system to achieve optimal spinal alignment by customizing regional mattress stiffness. Performance comparison of the active and non-active modes of the active-control mattress	Level of evidence: III 18 subjects (8 female, 10 male), age mean 31.3 ± 14.3 years. Field study: 12 subjects (6 female, 6 male), age mean 38.7 ± 23.4 years Sleeping posture: No control, postures were detected and estimated in system configuration; six sets of postures in a field study (supine, left/right lateral, prone, intermediate left/right) Mattress characteristics: Dynasleep mattress equipped with indentation sensors and adaptive actuator spring pockets Measurements: Spinal curvature Methods: indentation sensors embedded in Dynasleep mattress (spinal curvature was estimated using indentation data and a personalized human model)	The use of the active-control mattress system significantly improved the perceived sleep quality
Wu et al. [40]	Instrument design, repeated measurements	Development of a mattress evaluation method based on body pressure distribution and comparison of back surface and spinal alignment between supine lying and upright standing through finite element simulation. Comparison of the outcomes obtained for a palm fibre mattress and a bilayer latex/palm fibre mattress	Level of evidence: III 17 healthy subjects (4 female, 13 male), age mean 34.9 ± 9.7 years Sleeping posture: Supine Mattress type: 1. Palm fibre; (2) bilayer, upper layer: latex, lower layer: palm fibre, Young’s modulus *E *= 46.73 ± 5.72 kPa. Latex, hyper-elastic Ogden’s parameter, *m* = 1.28 ± 0.13 kPa, *a* = 4.175 ± 0.885, *b* = 0.314 ± 0.048 Measurements: (1) Back surface contour; (2) spinal alignment/mattress indentation; (3) body–mattress contact pressure Methods: (1) 3D body scanning system; (2) ANSYS finite element model; (3) Tactilus body pressure measurement system	A novel parameter was proposed by comparing the back surface contours of supine lying and natural standing postures via similarity analysis. The bilayer latex/palm fibre mattress produced a back surface contour close to that of upright standing, which indicated a preferable selection
Yoshida et al. [32]	Correlation (simulation versus subjective rating)	Investigation of the relationship between the outcome of computer simulation (finite element analysis) and subjective ratings on preference and comfort	Level of evidence: III 14 male college students aged 21–24 years. Finite element model: three subjects were picked from the pool to form the best body dimension coverage Sleeping posture: Supine Mattress type: Four types of pocket coil mattress with (1) *E* = 14.0 kPa; (2) *E* = 11.4 kPa; (3) *E* = 9.6 kPa; (4) *E* = 6.0 kPa Measurements: (1) internal stress, head and chest displacement; (2) subjective feedback Instruments: (1) ANSYS finite element model; (2) questionnaire, seven-grade scale on the feeling of firmness, mattress preference, firmness preference, sinking preference, comfort for different regions of the body	The subjective ratings corresponded to the prediction outcome, including the von Mises stress of the cervical vertebral region and the sinking displacement of the neck region
Zhong et al. [33]	Instrument design, validation (error analysis), mattress design process	Estimation of spinal curvature with mattress indentation Determination of an optimal mattress zonal stiffness	Level of evidence: III Nine females classified into three groups (*n* = 3) based on BMI Sleeping posture: Supine Mattress type: A total of 14 mattresses formed by the different combination of regional stiffness in five zones using six types of spring stiffness. The mattress consisted of a superficial layer of PU foam and a core layer composed of rows of pocketed springs Measurements: Spinal curvature Methods: (1) Custom-made indentation measuring bar embedded in the mattress (spinal curvature was estimated by fitting a curve on the indentation points)	The overall mean absolute error and mean relative error between the estimation and experimental measurements equalled 3.4 mm (SD 2.7) and 9.27%, respectively. cervicothoracic (CTh), thoracolumbar (ThL) and lumbosacral (LS) generally increased with lower back and hip zone stiffening; the upper body became more levelled with stiffened hip zones and more inclined with stiffened upper back zones

Level of evidence: 1 article was classified as evidence I (systematic review with and without meta-analysis); 7* as evidence II (randomized controlled clinical trial); 27* as evidence III (controlled clinical trial without randomization) and 4* as evidence level VI (evidence from a single descriptive or qualitative study). Each article has been carefully analysed by two independent reviewers to determine whether it was compliant in terms of inclusion and to evaluate methodological quality. In case of discrepancy between reviewers’ evaluations, a third reviewer intervened to analyse the controversy.

## Results

A total of 39 articles were considered compliant with inclusion criteria. Articles included in the current review and main results are presented in Table 1.

Such articles underlined the association between back pain decrease and mattress characteristics, in terms of design and firmness, that promote sleep quality and correct column alignment. Evaluating articles on effective pain relief deriving from use of specific mattresses, three crucial results emerged: three types of mattresses showed the ability to achieve more effective pain relief in study participants.

Some authors declared that a mattress with intermediate firmness might reduce back pain [6–10]. In the literature, some authors recommended mattresses with an air overlay system to reduce pain, while others showed that variation of temperature can promote sleep [11–14].

Jacobson et al. conducted a study comparing back pain, shoulder pain, spinal stiffness, quality, comfort and sleep efficiency in volunteers who had been usually sleeping on commercial spring mattresses (phase 1) and rested for 28 days on medium-firm mattresses (phase 2). Jacobson et al. used a pre-test and a post-test after the experimental phase, using symptomatic patients as a control. In all cases, independently from initial sleep control, benefits were observed as a result of using a medium-firm mattress, independently from age, weight, height, and body mass index (BMI). Besides, improvement appeared to be progressively increasing between the first and the fourth week since adoption of new mattresses [5].

Kovacs et al. conducted a double-blind multicentric controlled study evaluating 313 adults diagnosed with non-specific chronic low back pain upon waking up. Results showed that, even though improvement was observed using both mattresses, patients with medium-firm mattress reported a higher level of improvement both in terms of pain and disability. Thus medium-firm mattresses are recommended to patients suffering from non-specific chronic low back pain.

Some authors studied the efficacy of overlay systems made by using different techniques and materials. Specifically, Monsein et al. carried out a study on 30 patients diagnosed with severe low back pain who did not suffer from sleep disorders or sleep apnea syndrome.

Some authors studied the efficacy of overlay systems made by using different techniques and materials. Final results showed a significant benefit in terms of pain symptoms and sleep quality in patients who had slept on this kind of mattresses [11]. Similar results for what concerns pain decrease and sleep quality were reached by using an air-filled mattress low-pressure fixed overlay (Repose). A study from Price et al. was based on this type of overlay used for 4 weeks by 19 patients affected by chronic low back pain and sleep disorders. Results were statistically significant in terms of decrease in night awakenings as well as for sleep quality and relieved low back pain [12].

Body temperature is among parameters taken into consideration. Raymann group evaluated sleep quality in response to manipulating external body temperature by using a thermal suit with water perfusion and without altering core temperature. This study seems to be crucial to understanding the importance of mattress firmness; in fact, a mattress which is too firm does not let shoulders sink into the mattress, consequently leading to a lack of adequate support to neck and shoulders that causes pain and joint stiffness. On the other hand, in mattresses that are too soft, hips and shoulders sink too much into the mattress, leading to column misalignment. The study concluded that a customized mattress (customized inflation) is able to provide column with higher support during sleep on side position [4, 15]. Krauchi et al. in their study underlined once more the importance of room temperature in influencing sleep, even though it is still unclear how this occurs. Results showed an improved rachis alignment in beds adapted by an active control that adapted beds, supports and mattresses to the morphology of the examined rachis [16].

## Discussion

In the Jacobson et al. study, 59 healthy subject were enrolled: active individuals, free from known musculoskeletal pathologies, who had been sleeping on commercial spring mattresses for the past 5 years; however, they had occasionally reported physical discomfort during sleep, back stiffness once awake and poor sleep quality, even though no pathological condition associated with sleep had been diagnosed. Volunteers from this study were asked to fill a questionnaire daily for 28 days to investigate back pain, shoulder pain, column stiffness, quality, comfort and sleep efficiency [5, 7, 8]. The same questionnaires were administered to the same subjects during the following 28 days after having slept on a medium-firm mattress delivered at their households. The mattress had the following characteristics: medium-firm surface based on these components: foam-encased Bonnell spring unit, densified fibre pad, super-soft foam, damask cover, semi-flex foundation, slick fibred; they had the same size as mattresses previously used by volunteers. Results revealed significant improvement for all parameters previously indicated [14].

At the end of the experimental phase at day 28, a back pain decrease of approximately 48% and an improvement of sleep quality of 55% were reported; such improvement correlated with a significant decrease in stress levels.

At months 5–6 from the initial experimental phase, individuals were subjected to an additional assessment to complete the evaluation, reporting whether positive effects obtained had lasted over time. In addition, higher BMI was associated with worse sleep quality in both phases [8].

Kovacs et al. conducted a study with 313 adults with non-specific chronic low back pain upon waking up. Mattress firmness was scored based on the scale developed by the European Committee for standardization and went from 0 (maximum firmness) to 10 (minimum firmness). Firm (Hs = 2·3) and medium-firm (Hs = 5·6) mattresses were randomly allocated to patients. Patients were analysed at time 0 and at 90 days through a visual analogue scale (VAS) scale upon waking up and after 30 min and with a Spanish version of the Roland Morris questionnaire to evaluate the degree of disability experienced during daily activities.

In a study by Monsein et al., patients were asked to complete a Short Form 36 Health Survey Questionnaire (SF-36) and VAS scale at three different times: after sleeping in their own bed, after 28 days spent on a spring bed with an air topper and then after a period of sleep in their own beds again for 14 nights.

The majority of studies assessed mattress firmness on the basis subjective evaluations, except Kovacs et al., who applied the European Committee Standardization Scale of firmness of mattresses [9]. Subjects from all above-mentioned studies used a reproducible grading scale to assess back pain and sleep quality.

However, contrasting results can be found in the literature.

Several authors evaluating the relationship between mattress design and sleep quality studied materials and structural properties of spring mattresses [17]. No significant correlation was found between mattress firmness and sleep quality. However, authors described that a deeper and more effective sleep was observed with softer mattresses, having specificities related to the characteristics of each subject.

Tonetti et al. compared a latex mattress and a traditional spring mattress in 16 healthy volunteers, evaluating pre- and post-results of both through an actigraphy and a Mini Sleep Questionnaire (MSQ).

An objective improvement in sleep efficiency, sleep onset latency and motor activity during sleep was reported for both mattresses; however, no improvement in sleep quality subjective perception was highlighted for the latex mattress.

Likewise, Park et al. evaluated the correlation between anthropometric characteristics, body weight pressure distribution, spinal curve and characteristics of mattresses. In this study, six types of material were used for the mattresses: three different types of cotton, felt, sponge and elastic cotton. The column curve was measured both supinely and standing up through tridimensional measurements. Pressure measurements were taken by using sensors distributed from shoulders to hips. Patients were asked to classify each surface with a specific seven-point score for a subjective evaluation. Based on results from this study, the best mattress was that whose materials and design ensured a rachis curve similar to what usually showed while standing up [18].

Raymann group evaluated that an increase of just 0.4° led to a decrease in night awakenings and an improved sleep quality, also highlighted by an increase in slow wave activity recorded by electroencephalography [19]. This study was a pioneer for other studies to develop possible thermoregulation systems to be inserted inside a mattress.

Another parameter studied in the literature is the body–mattress contact pressure, commonly associated with pain level and discomfort [7, 20]. The system to measure body pressure was characterized by sheet sensors, so thin and flexible that they just minimally interfered with mattresses [21–24]. However, sensors can disperse concentrated pressure anyway and then underestimate the pressure peak. Similarly to the contact pressure principle, some studies analysed the loading effects of support in some areas using a load cell matrix. Peak pressure, mean pressure and contact area were often measured in different body sites to judge whether pressure was reduced. These tools were used by Fan-Zhe Low to demonstrate that, compared with polyurethane (PU) mattresses, latex mattresses better distribute body pressure points when lying down, reducing peaks involving thorax and sacral areas [25].

Spine alignment was the second most studied parameter, alongside body–mattress contact pressure. Physiological sagittal and coronal planes, in fact, reduce musculoskeletal pain. In the past, several studies carried out measurements of body alignment focusing on only two dimensions by using a video camera. Recently, some studies attempted to perform tridimensional (3D) measurements using an eye-tracking system through a webcam equipped with a depth sensor/infrared projector (Kinect, Microsoft, Redmond, WA, USA) to achieve a recording of images on both sagittal and coronal planes [15, 25–29], [30–33].

To evaluate sleep characteristics through objective data, some researchers identified a number of pressure points on the column that might be considered additional decisive factors to prescribe a mattress. Furthermore, patients were subjected to polysomnography and to a constant measurement of body temperature during sleep. Comfort was evaluated based on the body pressure on the mattress and on the column curvature both standing up and lying down. In this case, mattresses were evaluated as comfortable when during sleep they exhibited a curve with angles similar to the column curvature while standing up. As a result, the most comfortable mattresses that guaranteed also a better sleep quality were those that maintained a higher body temperature during sleep, at the same time supporting the column curve and minimizing unnecessary body movements [15, 34]. Based on findings achieved by studies previously illustrated, other research groups introduced a concept of “customized mattress” according to the posture of each subject.

In this study, soft, firm and customized mattresses were tested by applying sensors on participants’ spinous processes to record rachis alignment through an eye-tracking system during lateral position at rest for each of the three types of mattresses.

The objective was to evaluate how different types of mattresses modify rachis morphology in maximum pressure points in both supine and side positions. Mattresses used were: Perfect Contour Extraordinaire Dorchester By King Koil, Beautyrest Calibri Firm by Simmons; Posturepedic Afton Plush by Sealy; Perfect Sleeper Southdale by Serta. As expected, the pelvic area presented with higher values compared with the thorax. The best distribution of body contact pressure was attained by the Perfect Contour Extraordinaire Dorchester by King Koil, while The Perfect Sleeper Southdale by Serta showed a lower rachis curve alteration but higher contact pressure involving points of maximum pressure in both supine and prone positions [4].

Krauchi et al. used inclusion and exclusion criteria in their study. Twenty-eight male subjects aged between 25 and 30 years (BMI 19–25 kg/m^2^) were included in their study and evaluated through a sleep assessment chart. Exclusion criteria were: unsettled severe diseases, history of alcohol or illegal substances abuse, neurological disorders, cranial trauma and mental disorders according to the Diagnostic and Statistical Manual of Mental Disorders (DSM-V, American Psychiatric Association, 2013); other exclusion criteria were: Mini Mental Test Score < 26, administration of drugs affecting the central nervous system (CNS), excessive consumption of caffeine and/or tobacco (more than two cups and five cigarettes per day, respectively). All volunteers without anamnesis for sleep disturbance and normal sleep (Morningness-Eveningness Questionnaire: “none of the two types” [35]) were subjected to an in-hospital polysomnography (PSG) and a long-term monitoring of sleep–wake rhythm evaluated to exclude both sleep disorders (i.e. insomnia, sleep disorders caused by motor activity and or breathing disorders) and assess routine sleep duration and period of sleep. PSG was carried out using a portable monitoring device (Embletta MPR PG proxy ST+, Natus Medical Inc., Pleasanton, California, United States), four-channel electroencephalography (EEG) (C3, C4, O1, O2), two electrooculography chin and tibial electromyography, air flow nasal cannula, abdominal and thoracic breathing effort, oxygen saturation and heart rate, body position and snoring.

Routine sleep time, sleep onset latency, and sleep quality and efficiency were evaluated by wrist actigraphy for 2 weeks (software ActiGraph wGT3X and ActiLife + Sleep software, ActiGraph, Pensacola, FL, USA) together with sleep records. A high-heat-capacity mattress was tested in this study, in which foam layers at the bottom are covered by high-thermal-conductivity polyurethane layers (Technogel, Italia S.R.L., Vicenza, Italy), while the low-heat-capacity mattress is formed 100% by foam. For both mattresses, measures were 90 × 200 × 25 cm. The different thermal behaviour in HHCM and LHCM is correlated to the different density of the 2 cm covering the most superficial layer (HHCM, 1006 kg/m^3^; LHCM, 80 kg/m^3^) and then related to the different specific thermal capacity in the observed temperature interval of 23–35 °C (HHCM, about 47 kJ/°C; LHCM, about 5,4 kJ/°C for the 2 cm of the external surface). To exclude the potential effect of different overlays, the same type of overlay was used during the whole study (bi-elastic non-quilted fabric weighing 600 g/m^2^). This study also analysed temperature. To measure room, skin and mattress temperature, wireless temperature sensors were used (DS 1922L, Thermochron iButtons; Maxim, Dallas, USA; resolution 0.0625 °C; sampling frequency one value per minute).

Video polysomnography was performed by Comet XL Lab-based PSG (Grass Telefactor, Astro-Med Inc., West Warwick, RI 02893 USA) using four-channel EEG (C3, C4, O1, O2), two electrooculogram channels (right and left outer canthus), chin electromyogram, heart rate, oxygen saturation and body position.

Temperature in the room was kept around 23 °C with a ± 0.5 °C interval and relative humidity between 45% and 55%. PSG variables included: time spent in phases N1, N2, N3 (slow wave sleep, SWS), rapid eye movement (REM) sleep, total sleeping time, sleep efficiency and time of wake-up after sleep onset.

The most important result achieved by this study is that comparison between individuals sleeping on low-thermal-capacity mattresses (LHCM) and high-thermal-capacity mattresses (HHCM) demonstrated a significant reduction in core body temperature (CBT), proximal skin temperature on the back (PROBA) and mattress surface temperature, and a significant increase in N3 sleep phase. Regression analysis selectively revealed a significant association between increased CBT-PROBA (and reduced PROBA) with emphasized N3 sleep phase. The present study conducted in a controlled laboratory demonstrated that sleep characteristics can be influenced by thermal properties of the mattress. Comparison between a traditional mattress LHCM and HHCM mattress showed an increase in temperature of proximal skin on the back and of the core body temperature, and an increase in slow sleep waves and sleep continuity [13].

## Conclusions

Based on data reported by literature, it can be claimed that medium-firm mattresses offer more advantages to subjects with non-specific low back pain. Studies have demonstrated, indeed, that these mattresses improve sleep quality and reduce risk of developing low back pain. Beds with active control improve column alignment and sleep quality. Temperature manipulation using a HHCM mattress caused a reduction in temperature increase of proximal skin on the back and of core body temperature, and an increase in slow sleep waves and sleep continuity; slightly manipulating the skin temperature, the wake-up can be delayed and deep sleep can be favoured.

## Limitations

The articles analysed reported differed with regard to their observations, and standardization of volunteers was low in terms of physical and health characteristics. Type of low back pain was poorly determined.

## Data Availability

Studies carried out between 2000 and 2019 have been included in the current review. Mattresses analysed in the studies examined by our research group have been classified according to the European Committee for Standardization (2000) as soft, medium-firm, extra-firm or customized to avoid supine decubitus. All authors read and approved the final manuscript.
